# Immunogenicity of Low-Dose Prime-Boost Vaccination of mRNA Vaccine CV07050101 in Non-Human Primates

**DOI:** 10.3390/v13081645

**Published:** 2021-08-19

**Authors:** Neeltje van Doremalen, Robert J. Fischer, Jonathan E. Schulz, Myndi G. Holbrook, Brian J. Smith, Jamie Lovaglio, Benjamin Petsch, Vincent J. Munster

**Affiliations:** 1 Laboratory of Virology, National Institute of Allergy and Infectious Diseases, National Institutes of Health, Hamilton, MT 59840, USA; neeltje.vandoremalen@nih.gov (N.v.D.); fischerro@niaid.nih.gov (R.J.F.); jonathan.schulz@nih.gov (J.E.S.); myndi.holbrook@nih.gov (M.G.H.); 2 Rocky Mountain Veterinary Branch, National Institute of Allergy and Infectious Diseases, National Institutes of Health, Hamilton, MT 59840, USA; brian.smith2@nih.gov (B.J.S.); jamie.lovaglio@nih.gov (J.L.); 3 CureVac AG, 72076 Tuebingen, Germany; benjamin.petsch@curevac.com

**Keywords:** SARS-CoV-2, CureVac, COVID, vaccine, NHP

## Abstract

Many different vaccine candidates against severe acute respiratory syndrome coronavirus 2 (SARS-CoV-2), the etiological agent of COVID-19, are currently approved and under development. Vaccine platforms vary from mRNA vaccines to viral-vectored vaccines, and several candidates have been shown to produce humoral and cellular responses in small animal models, non-human primates, and human volunteers. In this study, six non-human primates received a prime-boost intramuscular vaccination with 4 µg of mRNA vaccine candidate CV07050101, which encodes a pre-fusion stabilized spike (S) protein of SARS-CoV-2. Boost vaccination was performed 28 days post prime vaccination. As a control, six animals were similarly injected with PBS. Humoral and cellular immune responses were investigated at time of vaccination, and two weeks afterwards. No antibodies could be detected at two and four weeks after prime vaccination. Two weeks after boost vaccination, binding but no neutralizing antibodies were detected in four out of six non-human primates. SARS-CoV-2 S protein-specific T cell responses were detected in these four animals. In conclusion, prime-boost vaccination with 4 µg of vaccine candidate CV07050101 resulted in limited immune responses in four out of six non-human primates.

## 1. Introduction

Severe acute respiratory syndrome coronavirus 2 (SARS-CoV-2) is the etiological agent responsible for COVID-19. SARS-CoV-2 has spread worldwide, and over 185 million cases were detected as of July 2021. The pandemic resulted in an unprecedented research effort towards the development of a SARS-CoV-2 vaccine, and several vaccines against SARS-CoV-2 have now been approved. Interestingly, whilst traditional approaches such as subunit protein vaccines [1] and inactivated virus vaccines [2] are still pursued, a large number of vaccines are based on novel platforms such as virus-vectored vaccines [3,4,5] and nucleic acid (DNA or RNA) vaccines [6,7]. Promising results have been published for these platforms, both preclinical [8,9,10,11,12,13] and clinical [3,4,5,6,7], showing the induction of a humoral and cellular response. 

Preclinical assessment of SARS-CoV-2 vaccines in non-human primate models is advantageous due to the close relatedness of non-human primates to humans, thereby resulting in a higher degree of clinical translation than smaller animal models. Indeed, rhesus macaques have been successfully used to study vaccines [14]. Inoculation of rhesus macaques with SARS-CoV-2 results in respiratory disease, which includes virus replication in upper and lower respiratory tract [15]. Two reports on the immune response of SARS-CoV-2 mRNA vaccine candidates in non-human primates describe the induction of binding and neutralizing antibodies, as well as antigen-specific T cell responses [9,10]. 

SARS-CoV-2 messenger RNA (mRNA) vaccines encoding the SARS-CoV-2 spike (S) protein have a good safety and immunogenicity profile, both in non-human primates [9,10] and in humans [6,7,16]. Here, we investigate the immunogenicity of another SARS-CoV-2 S mRNA vaccine, CV07050101, in non-human primates. CV07050101 is based on mRNA technology, RNActive^®^, developed by CureVac for the accelerated development of human vaccines [17,18,19,20,21]. The efficaciousness of this platform has been demonstrated for a rabies vaccine in mice and humans [18,22]. Moreover, mRNA vaccines have been discussed as particularly well suited to combating outbreak pathogens [23].

## 2. Materials and Methods

### 2.1. Ethics Statement

Animal study approval was provided by the Institutional Animal Care and Use Committee (IACUC) at Rocky Mountain Laboratories. Animal experiments were conducted in an AAALAC-approved facility, following the basic principles and guidelines in The Guide for the Care and Use of Laboratory Animals, the Animal Welfare Act, United States Department of Agriculture and the United States Public Health Service Policy on Humane Care and Use of Laboratory Animals. Rhesus macaques were housed in individual primate cages allowing social interactions, in a climate-controlled room with a fixed light/dark cycle (12 h/12 h). Animals were monitored at least twice daily and commercial monkey chow, treats, vegetables, and fruit were provided. Water was available ad libitum. A variety of human interaction, commercial toys, videos, and music was used as environmental enrichment. 

### 2.2. Vaccine mRNA and Lipid Nanoparticle Production

CV07050101 is a lipid-nanoparticle-formulated RNActive^®^ SARS-CoV-2 vaccine composed of the active pharmaceutical ingredient, an mRNA that encodes a pre-fusion conformation-stabilized version of the full-length spike (S) protein of SARS-CoV-2 virus (GenBank YP_009724390.1), including the K986P and V987P prefusion stabilizing mutations, and four lipid components: cholesterol, 1,2-distearoyl-sn-glycero-3-phosphocholine (DSPC), PEGylated lipid, and a cationic lipid [24].

### 2.3. Study Design

Twelve male rhesus macaques 3–5 years old were screened for SARS-CoV-2 status by ELISA, and when found to be negative for prior exposure were sorted by body weight and divided into two groups of six animals, resulting in near equal contribution of body weights. Group 1 (vaccine) was vaccinated with 4 µg of mRNA vaccine CV07050101 in sterile PBS at 0 and 28 days. Group 2 (control) was vaccinated with sterile PBS at 0 and 28 days via intramuscular injection, using Monoject 1 mL Tuberculin syringes (Covidien, 25G x 5/8″). Blood samples were obtained before vaccination and 14 days after each vaccination. Hematology analysis was completed on a ProCyte DX (IDEXX Laboratories, Westbrook, ME, USA), and the following parameters were evaluated: red blood cells (RBC), hemoglobin (Hb), hematocrit (HCT), mean corpuscular volume (MCV), mean corpuscular hemoglobin (MCH), mean corpuscular hemoglobin concentration (MCHC), red cell distribution weight (RDW), platelets, mean platelet volume (MPV), white blood cells (WBC), neutrophil count (abs and %), lymphocyte count (abs and %), monocyte count (abs and %), eosinophil count (abs and %), and basophil count (abs and %). Serum chemistries were completed on a VetScan VS2 Chemistry Analyzer (Abaxis, Union City, CA, USA) and the following parameters were evaluated: glucose, blood urea nitrogen (BUN), creatinine, calcium, albumin, total protein, alanine aminotransferase (ALT), aspartate aminotransferase (AST), alkaline phosphatase (ALP), total bilirubin, globulin, sodium, potassium, chloride, and total carbon.

### 2.4. Enzyme-Linked Immunosorbent Assay

A plasmid encoding the prefusion stabilized SARS-CoV-2 spike protein with a T4 fibritin trimerization motif was obtained from the Vaccine Research Centre, Bethesda, MD, USA and expressed in-house. Maxisorp plates (Nunc, Roskilde, Denmark) were coated overnight at 4 °C with 100 ng/well spike protein in PBS. Plates were blocked with 100 µL of casein in PBS (Thermo Fisher, Rockville, MD, USA) for 1 h at RT. Serum serially diluted 2× in casein in PBS was incubated at RT for 1 h. Antibodies were detected using affinity-purified polyclonal antibody peroxidase-labeled goat-anti-monkey IgG (Seracare, Milford, MA, USA, 074-11-021) in casein and TMB 2-component peroxidase substrate (Seracare, Milford, MA, USA, 5120-0047), developed for 5–10 min, and reaction was stopped using stop solution (Seracare, Milford, MA, USA, 5150-0021) and read at 450 nm. All wells were washed 3× with PBST 0.1% Tween between steps. Threshold for positivity was set at 3× OD value of negative control (serum obtained from non-human primates prior to start of the experiment) or 0.2, whichever was higher.

### 2.5. ELISpot

PBMCs were isolated from ethylene diamine tetraacetic acid (EDTA) whole blood using LeucosepTM tubes (Greiner Bio-one International GmbH, Frickenhausen, Germany) and Histopaque^®^-1077 density gradient cell separation medium (Sigma-Aldrich, St Louis, MO, USA) according to the manufacturers’ instructions. IFN-γ ELISpot assay of PBMCs was performed using the ImmunoSpot^®^ Human IFN-γ Single-Color Enzymatic ELISpot Assay Kit according to the manufacturer’s protocol (Cellular Technology Limited, Shaker Heights, OH, USA). PBMCs were plated at a concentration of 300,000 cells per well and were stimulated with two contiguous peptide pools spanning the length of the SARS-CoV-2 S protein sequence at a concentration of 2 µg/mL per peptide (Mimotopes, Mulgrave, Australia). Imaging was performed using the CTL ImmunoSpot^®^ Software (Cellular Technology Limited, Shaker Heights, OH, USA). Spot forming units (SFU) were hand counted and calculated per 10 [6] PBMCs as summed across the peptide pools for each animal.

### 2.6. SARS-CoV-2 Virus Neutralization

VeroE6 cells were maintained in Dulbecco’s modified Eagle’s media (DMEM) supplemented with 10% fetal bovine serum (Gibco, Amarillo, TX, USA), 1 mM L-glutamine, 50 U/mL streptomycin, and 50 ug/mL penicillin. Sera were heat-inactivated (30 min, 56 °C); two-fold serial dilutions were prepared in DMEM supplemented with 2% fetal bovine serum (Gibco), 1 mM L-glutamine, 50 U/mL streptomycin, and 50 ug/mL penicillin; and 100 TCID_50_ of SARS-CoV-2 was added. After 1 h incubation at 37 °C and 5% CO_2_, virus–serum mixture was added to VeroE6 cells and incubated at 37 °C and 5% CO_2_. At 6 dpi, cytopathic effect was scored. The virus neutralization titer was expressed as the reciprocal value of the highest dilution of the serum, which still inhibited 100% of virus replication. A positive control standardized against the NIBSC serum control 20/130 was used in all VN assays.

## 3. Results

**Figure 1:**
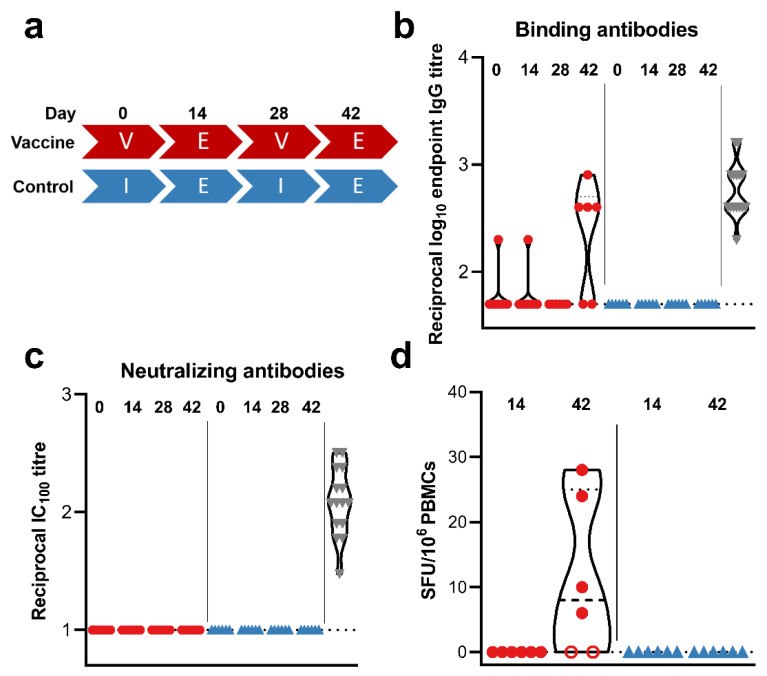
(**a**) Study schedule. Two groups (N = 6) were vaccinated (V) or administered PBS (I) twice, four weeks apart. Fourteen days post each vaccination, exams (E) were performed. The presence of SARS-CoV-2 spike-specific binding (**b**) and neutralizing (**c**) antibodies in serum obtained from rhesus macaques at time of vaccination and 14 days afterwards were measured using ELISA and infectious virus neutralization assays. (**d**) SARS-CoV-2 S-specific T cell responses were measured via ELIspot. Closed red circles = animal positive in ELISA assay; open red circle = animal negative in ELISA assay; blue triangles = control animals; grey triangles = convalescent human sera; dotted line = lower limit of detection.

In order to investigate the immunogenicity of mRNA vaccine CV07050101, we vaccinated six rhesus macaques (all male) at 0 and 28 days via intramuscular injection, using 4 µg per dose. We chose a dose of 4 µg in rhesus macaques since this is comparable to 12 µg in humans, the dose used in the Phase III clinical trial [25]. As a control, six rhesus macaques were injected with an equal volume of sterile PBS (Figure 1a). No adverse events were observed upon vaccination, and overall hematology and clinical chemistries were unremarkable. No differences between the control and vaccinated groups were noted. No binding antibodies could be detected 14 or 28 days post prime vaccination (Figure 1b). At 14 days post boost vaccination, low titers of spike-specific binding antibodies (reciprocal endpoint IgG titers of 400–800) could be detected in four out of six animals (Figure 1b). Virus-specific neutralizing antibodies were not detected in animals at any time post boost vaccination (Figure 1c). No SARS-CoV-2 spike-specific T cell responses were detected 14 days post prime vaccination but were detected in the same four out of six animals at 14 days post boost vaccination (Figure 1d). The detection of specific T cell responses correlated with the detection of spike-specific binding antibodies. 

## 4. Discussion

Here we show that the prime-boost vaccination of rhesus macaques with 4 µg of CV07050101 resulted in the induction of binding antibodies in some, but not all, vaccinated animals. This contrasts with other studies of mRNA vaccines, in which a prime-boost vaccination elicits a robust humoral and cellular response in all animals. Using a prime-boost regimen of 10 µg of mRNA-1273, which encodes a prefusion-stabilized S protein utilizing modified mRNA, S-specific binding antibodies were detected in all animals, whereas neutralizing antibodies were detected in seven out of eight animals [9]. Likewise, a prime-boost vaccination using 30 µg of vaccine candidate BNT162b2, which also encodes a prefusion-stabilized S protein, elicited binding and neutralizing antibodies in six out of six animals. Moreover, binding and neutralizing antibodies were detected in all animals 21 days post prime-only vaccination with 30 µg of BNT162b2 [10]. A recently released preprint investigating CV07050101 showed that the vaccination of rhesus macaques with 8 µg of mRNA elicited binding and neutralizing antibodies, whereas a dose of 0.5 µg of mRNA did not [26]. 

One important difference between these studies is the amount of mRNA used to vaccinate animals. We used 4 µg of mRNA per vaccination, whereas the other doses which elicited an immune response used between 8 µg and 100 µg per vaccination [9,10,24]. Using a dose of 10 µg mRNA-1273 vaccine resulted in no detectable neutralizing antibodies in one out of eight animals on the day of challenge, but 100 µg of mRNA-1273 resulted in neutralizing antibodies in all animals in [9], suggesting a dose-dependent response to the vaccine. Likewise, whereas 8 µg of CV07050101 induced an immune response, 0.5 µg did not in [26]. 

Compared to the limited immunogenicity in non-human primates we observed here, robust SARS-CoV-2-neutralizing titers were observed in BALB/c mice immunized with a prime-boost regimen of 2 µg of the CV07050101 vaccine. Challenge studies in hamsters, which were performed at a later stage, utilized a 10 µg prime-boost regimen of CV07050101 vaccine and a challenge dose of 100 TCID_50_ SARS-CoV-2 and provided protection of the lower respiratory tract [24]. Using allometric scaling of the vaccine dose to the human equivalent dose [25] results in an mRNA vaccination with 24.6 µg (mouse study), 74 µg (hamster study), and 12.2 µg (current study). Thus, it is difficult to directly compare results between the studies. 

As the elicited immune response was low or absent in the vaccinated rhesus macaques, we decided not to challenge the animals. In rhesus macaques, neutralizing antibodies are a correlate of protection [27]. The presence of neutralizing antibodies in humans correlates with immunity against SARS-CoV-2 [28]. Since we did not detect neutralizing antibodies, we hypothesize that these animals would not have been protected. 

Since CV07050101 is now assessed in clinical trial studies, we compared the available immunogenicity results. The 12 µg high-dose vaccine prime-boost regime was able to induce neutralizing antibody titers comparable to non-hospitalized individuals, whereas the 2–8 µg doses induced neutralizing titers that were lower in clinical trial participants. However, virus-neutralizing antibodies could be detected in 66% of human volunteers given 4 µg of CV07050101 in [16], in contrast to a lack of neutralizing antibodies in serum from NHPs vaccinated with the same dose. It has been hypothesized that a difference in the lubricant used in syringes can decrease the integrity of mRNA vaccines, which may explain the low immunogenicity detected in this NHP study (B.P. personal communication). 

To improve our understanding of vaccine development, it is crucial to understand why certain vaccines are superior to others. The importance of the usage of unmodified mRNA (CureVac) versus modified mRNA (Pfizer, Moderna) should be further investigated by vaccinating animals with the exact same vaccine using the same dose but using either modified or unmodified mRNA. Furthermore, a study performed by one research group investigating the three leading mRNA vaccines in the same animal model at the same dose would inform us on how important the dose of mRNA is. 

In conclusion, we show that prime-boost vaccination of rhesus macaques with 4 µg of CV07050101 elicited neither a uniform nor a robust immune response. However, vaccination using 8 µg of the same vaccine was protective in an NHP challenge study demonstrating protection against SARS-CoV-2 infection by CV07050101 vaccination [26]. 

## Data Availability

All data is available on doi:10.6084/m9.figshare.15208539.
